# Supplementary material for: Association between oral microbiome and seven types of cancers in East Asian population: a two-sample Mendelian randomization analysis
Source: Front Mol Biosci. 2023 Nov 21;10:1327893. doi: 10.3389/fmolb.2023.1327893 (PMC10702768; doi:10.3389/fmolb.2023.1327893)
Supplement: Supplementary file 1 [file DataSheet1.ZIP › Supplementary Materials/MR plots for tongue/tongue═╝/Pancreatic cancer/╥╚╧┘░⌐_╔╕╤í╜ß╣√/pheno.2192_to_pancreatic cancer_leave_one_out.pdf]

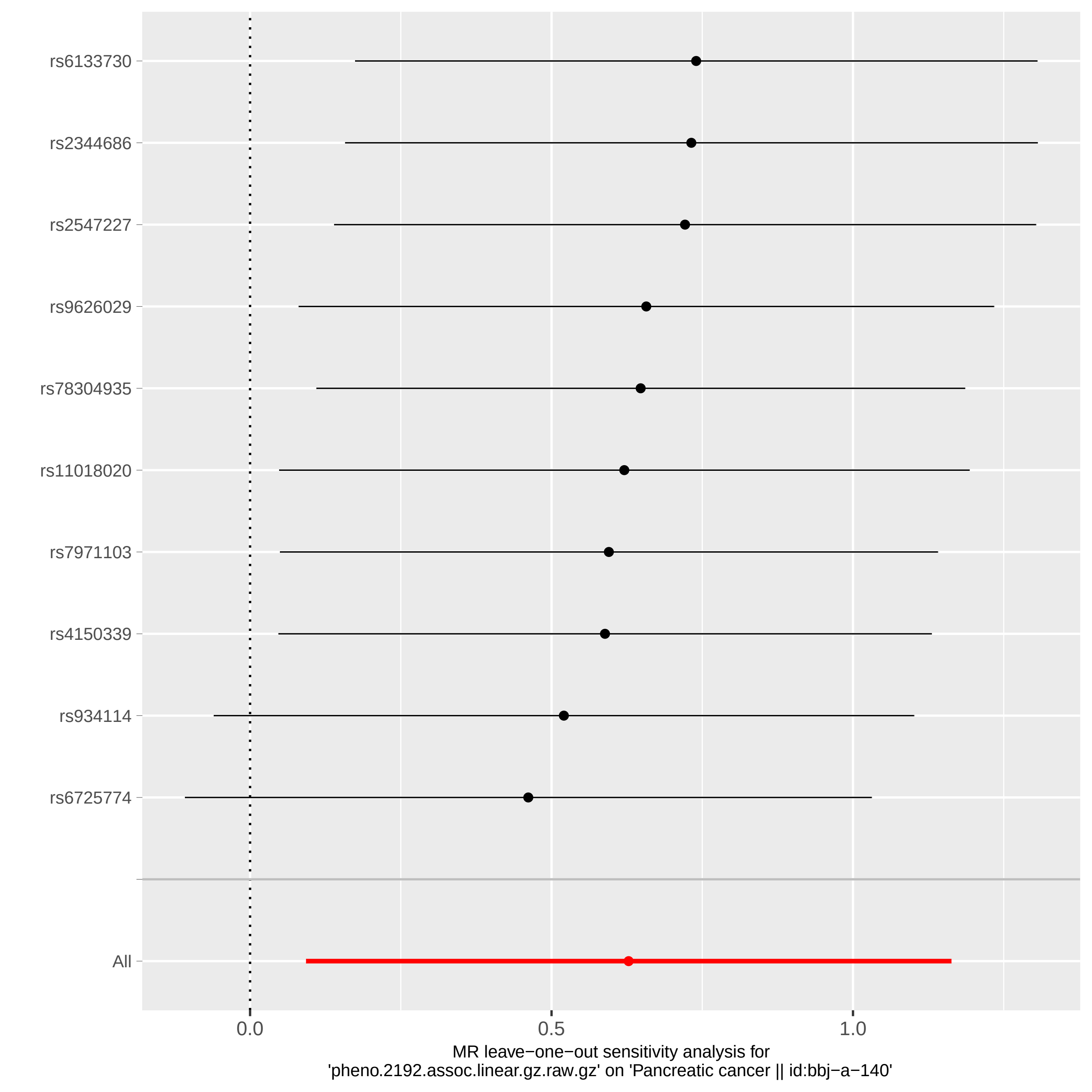
rs6133730
rs2344686
rs2547227
rs9626029
rs78304935
rs11018020
rs7971103
rs4150339
rs934114
rs6725774
All
0.0
0.5
1.0
MR leave−one−out sensitivity analysis for
'pheno.2192.assoc.linear.gz.raw.gz' on 'Pancreatic cancer || id:bbj−a−140'
